# β-Cyclodextrin Nanosponges Inclusion Compounds Associated with Silver Nanoparticles to Increase the Antimicrobial Activity of Quercetin

**DOI:** 10.3390/ma16093538

**Published:** 2023-05-05

**Authors:** Sebastián Salazar Sandoval, Tamara Bruna, Francisca Maldonado-Bravo, Karen Bolaños, Sofía Adasme-Reyes, Ana Riveros, Nelson Caro, Nicolás Yutronic, Nataly Silva, Marcelo J. Kogan, Paul Jara

**Affiliations:** 1 Departmento de Química, Facultad de Ciencias, Universidad de Chile, Las Palmeras 3425, Ñuñoa, Santiago 7610658, Chile; 2 Departamento de Química Farmacológica y Toxicológica, Universidad de Chile, Sergio Livingstone 1007, Santiago 8380492, Chile; scadasmer@gmail.com (S.A.-R.); riveros.ana@gmail.com (A.R.);; 3 Advanced Center for Chronic Diseases (ACCDiS), Universidad de Chile, Santos Dumont 964, Independencia, Santiago 8380494, Chile; k.bolaosjimenez@uandresbello.edu; 4 Facultad de Diseño, Universidad del Desarrollo, Avenida Plaza 680, Las Condes, Santiago 7610658, Chile; 5 Centro de Investigación Austral Biotech, Facultad de Ciencias, Universidad Santo Tomás, Avenida Ejército 146, Santiago 8320000, Chile; 6 Laboratory of Cellular Communication, Program of Cell and Molecular Biology, Center for Studies on Exercise, Metabolism and Cancer (CEMC), Institute of Biomedical Sciences (ICBM), Facultad de Medicina, Universidad de Chile, Av. Independencia 1027, Santiago 8380453, Chile

**Keywords:** β-cyclodextrin-based nanosponges, antimicrobials, quercetin, silver nanoparticles, plasmon surface resonance, antimicrobial activity

## Abstract

This work aimed to synthesize and characterize a nanocarrier that consisted of a ternary system, namely β-cyclodextrin-based nanosponge (NS) inclusion compounds (ICs) associated with silver nanoparticles (AgNPs) to increase the antimicrobial activity of quercetin (QRC). The nanosystem was developed to overcome the therapeutical limitations of QRC. The host–guest interaction between NSs and QRC was confirmed by field emission scanning electron microscopy (FE–SEM), X-ray powder diffraction (XRPD), thermogravimetric analysis (TGA), and proton nuclear magnetic resonance (^1^H–NMR). Moreover, the association of AgNPs with the NS–QRC was characterized using FE–SEM, energy-dispersive spectroscopy (EDS), transmission electron microscopy (TEM), dynamic light scattering (DLS), ζ-potential, and UV–Vis. Finally, the antimicrobial activity of the novel formulations was tested, which depicted that the complexation of QRC inside the supramolecular interstices of NSs increases the inhibitory effects against *Escherichia coli* ATCC25922, as compared to that observed in the free QRC. In addition, at the same concentrations used to generate an antibacterial effect, the NS–QRC system with AgNPs does not affect the metabolic activity of GES–1 cells. Therefore, these results suggest that the use of NSs associated with AgNPs resulted in an efficient strategy to improve the physicochemical features of QRC.

## 1. Introduction

Cyclodextrins (CDs) are a family of water-soluble and cyclic oligosaccharides with a hydrophilic outer surface and a hydrophobic central cavity. Among them, β-CDs are remarkable due to their cavity dimensions (7.8 A), which are appropriate to accommodate benzene compounds or species with similar structures [1,2,3]. As such, β-CDs are very valuable in drug formulations to improve the physicochemical properties of the actives, namely bioavailability, solubility, and stability [4,5]. β-CDs have also been used as building blocks and monomers for developing new drug carriers, such as β-CD nanosponges (NSs) [6,7,8]. NSs display high thermal stability, encapsulation efficiencies, tuneable pore sizes, and control over their solubility, cross-linking, and solubility [9,10,11,12,13,14,15]. In this context, NSs are usually preferred over native CDs in pharmaceutical applications to overcome drug delivery barriers, with the drug release rate, degradation, and permeability being the most concerning.

Many molecules have been studied concerning their potential uses as anti-tumour drugs or antimicrobials. With this precedent, flavonoids have shown promising results as bactericide agents. Quercetin (3,3′,4′,5.7-pentahydroxyflavone) is a flavonoid compound widely present in plants, exhibiting antioxidant and antibacterial properties. Despite its broad biological properties, quercetin (QRC) use in pharmaceutics is minimal due to its low aqueous solubility and photodegradation [16,17,18]. Regarding these disadvantages, NSs can form inclusion compounds through host–guest interactions with different molecules [19,20,21]. The interstitial sites formed in the cross-linking reaction can act as supramolecular sites for the entrapment of drugs. As such, the formation of an inclusion complex can improve the stability and aqueous solubility of QRC.

Furthermore, the association of the inclusion complexes with nanoparticles can provide the systems with additional and valuable properties. Among the nanoparticles that have been deposited in the organic matrices, gold and magnetite stand out. Magnetite nanoparticles associated with NSs have been used to remove pollutants from aqueous media while improving the adsorbent’s reusability and recyclability [22,23,24,25]. Moreover, gold nanoparticles and anisotropic gold nanoparticles have been stabilized with NS inclusion complexes to develop a drug delivery system utilizing local photothermia [26,27,28,29].

Considering this, silver nanoparticles (AgNPs) also come to mind when proposing a new ternary system. AgNPs, like their AuNP counterparts, exhibit unique optical properties, which also could have a potential application in drug delivery systems. However, the property of AgNPs that recently has been the focus of research is their antibacterial activity [30,31,32]. AgNPs have exhibited antimicrobial activity against multiple infectious microorganisms. As such, the antibacterial activity of AgNPs is a valuable tool to incorporate in a formulation where a synergic effect might occur. AgNPs can be stabilized and associated with organic substrates if the latter presents amines, hydroxyls, thiols, or carboxyl in its molecular structure [33]. NS inclusion compounds associated with AgNPs might have superior antimicrobial activity compared to the free species.

**Figure 1:**
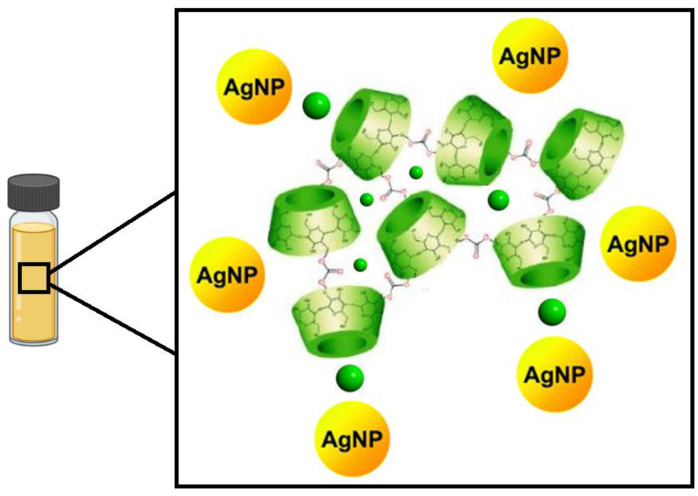
Schematic representation of the ICs associated with AgNPs. The antimicrobial QRC is represented in green circles.

This study describes the formation of inclusion compounds consisting of NSs and an antimicrobial guest, namely QRC. The functional groups on the inclusion compound provided stability to the AgNPs and allowed the formation of ternary systems consisting of AgNP–NS–QRCs (Figure 1). The physicochemical characterization of the ICs was carried out to confirm the inclusion of the guests. In contrast, the ternary systems were also analyzed to confirm the immobilization of AgNPs onto the organic polymer. Finally, analyses were performed to determine the antimicrobial activity of the novel composites.

An antimicrobial system consisting of NSs inclusion compounds associated with AgNPs has not been reported to date, which could be promising as a potential technology to combat pathogen bacteria. As such, the objectives of this work can be summarized as follows:

1. The development of an NSs–QRC formulation to improve the stability of free QRC.
2. The association of AgNPs to the NSs–QRC complex to generate the AgNPs–NSs–QRC ternary system and the further evaluation of its antimicrobial properties.
3. Evaluating the biocompatibility and biotoxicity of the NS–QRC and AgNPs–NSs–QRC complexes.

## 2. Materials and Methods

### 2.1. Materials

Anhydrous β-cyclodextrin, C_42_H_70_O_35_, ≥97%, 1134.98 g/mol; quercetin, C_15_H_10_O_7_, 95%, 302.2 g/mol; diphenyl carbonate, C_6_H_5_O, 99%, 214.2 g/mol; silver nitrate, AgNO_3_, ≥99.9%, 169.8 g/mol; sodium borohydride, NaBH_4_, ≥99%, 37.83 g/mol; oleic acid, C_18_H_34_O_2_, ≥99.9%, 282.46 g/mol, density (δ): 0.89 g/cm^3^; and nano-pure water were obtained from Merck (Merck, Darmstadt, Germany) and used with no further purification. Aqua regia (3 HCl:1 HNO_3_) and Milli–Q water were used to wash all glassware to synthesize nanomaterials. Cell culture-treated plates and flasks were purchased from Falcon. Penicillin/streptomycin (Thermo Fisher Scientific, Waltham, MA, USA), FBS (Biological industries, Göttingen, Germany) (3-(4,5-dimethylthriazol- 2-yl)-5-(3-carboxymethoxyphenyl)-2-(4-sulfophenyl)- 2H-tetrazolium inner salt) (MTS) and phenazine methosulfate (PMS) were purchased from Promega (Madison, WI, USA). Other reagents were purchased from Thermo Scientific.

### 2.2. Synthesis of AgNPs

As reported by previous protocols, the colloidal AgNPs were synthesized using NaBH_4_ as the reducing agent and oleic acid as the capping agent [34]. Briefly, 25 mL of a 1.7 mM solution of AgNO_3_ was mixed with 52.8 μL of a 3 mM solution of oleic acid and stirred vigorously at 25 °C. Then, 25 mL of a NaBH_4_ 30 mM solution were added gradually to the mixture, thus reducing the Ag^+^ ions. The solution turned to a brownish yellow after adding the reducing agent.

### 2.3. Synthesis and Purification of the NSs Matrix

The NSs were obtained according to previously reported methods [35], with minor modifications to reduce the by-products and trace precursors. Anhydrous β-CD (1.5 g) and diphenylcarbonate (0.85 g) were homogenized and placed in an Erlenmeyer flask. Subsequently, the mixture was heated to 100 °C and stirred for 5 h. Afterward, the solid was cooled at room temperature and further grounded with an agate mortar. To separate the unreacted precursors from the obtained NSs, the product was washed thoroughly with double distilled water and acetone.

**Figure 2:**
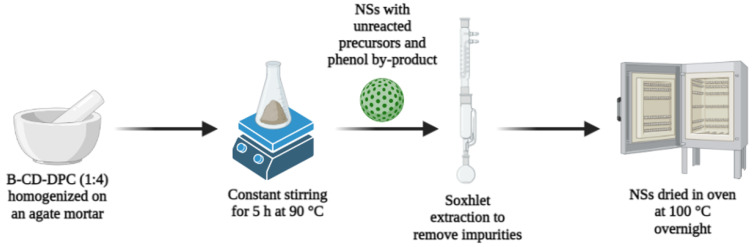
Schematic representation of the melting method used to synthesize the NSs. Created with Biorender.com.

Soxhlet extraction removed the phenol by-product formed by the cross-linking reaction, using ethanol and acetone as solvents. After drying the NSs at 100 °C for 48 h, the polymer was stored in a desiccator for further use. Figure 2 describes the synthetic route used to obtain the NSs.

### 2.4. Formation of the NSs-QRC Inclusion Compound

The NSs were loaded with QRC using previously reported methods [11,12]. Twenty milligrams of NSs were dispersed in 50 mL of Milli–Q water at room temperature and kept under vigorous agitation. Further, 20 mL of a solution of QRC was added to the suspended NSs. After sonication for 10 min. and stirring for 1 day, the obtained NS–QRC complex was centrifuged and separated from the guests that were not part of a complex. Finally, the inclusion compounds (ICs) were lyophilized and stored at room temperature.

### 2.5. Formation of the ICs–AgNPs Ternary Complexes

The IC–AgNPs ternary complexes were formed by adding 30 mg of NSs–QRC into 1 mL of AgNPs. After 20 min, the systems were centrifuged at 20,000 rpm for 30 min. The ICs turned from white to yellow upon association with the AgNPs. After separating the ternary complex from the supernatant, they were washed and resuspended in a new aqueous solution to eliminate the AgNPs that were not in a complex. Atomic absorption spectroscopy (AAS) was used to determine the concentration of the AgNPs that were deposited in the organic matrix.

### 2.6. Characterization Methods

^1^H–NMR spectroscopy was used to evaluate the host–guest interaction between the NSs and the antimicrobial. A Bruker Advance 400 MHz spectrometer (Bruker, Billerica, MA, USA) at 300 K was used with tetramethylsilane (TMS) as an internal reference. Twenty milligrams of each sample were dissolved using 0.6 mL of deuterated dimethyl sulfoxide (DMSO–d_6_). The Mestre Nova program was used to analyze and determine the chemical shifts in the ^1^H-NMR spectrum.

The topographic and surface information of NSs, QRC, and the NS-guest complexes were obtained using a Zeiss LEO Supra 35–VP scanning electron microscope (Oberkochen, Germany) with an acceleration voltage of 20 kV. The samples were deposited directly onto carbon tapes, and a magneton sputtering was used for gold coating. The elemental composition of the abovementioned systems was provided by energy dispersive spectroscopy (EDS).

XRPD diffractograms of NSs, QRC, and the NS-guest complexes were provided by a Siemens/Bruker D5000 diffractometer (Billerica, MA, USA). The system is equipped with a Cu anode X-ray tube and Ni target filter. A 40 kV/40 mA current was used, with a scan speed of 0.05°/s. The analysis of the samples was performed over a 2° < 2ϴ < 50° angle range.

TGA of the NSs, QRC, and the NS-guest complexes was performed on a TGA–4000 Pyris 6 over a 25–600 °C temperature range. Each sample, at 10 mg, was placed and weighted on an aluminum pan. All measurements were conducted under nitrogen atmosphere.

UV–Visible spectroscopy was performed to ascertain the presence of the characteristic plasmon band of AgNPs and the ternary systems. A Jasco V–760 UV–Visible spectrometer (Madrid, Spain) was used to record 200–800 nm measurements. Milli–Q water was used as a baseline, whereas the spectra were processed using UVProve ver. 2.70 software.

Particle size and morphology of AgNPs and the ternary systems were analyzed through TEM. The micrographs were obtained using a Hitachi model HT–7700 microscope at 120 kV. The AgNPs and the ternary systems were subjected to sonication, then 20 μL of each sample were dropped directly onto a copper grid with a Formvar film.

The hydrodynamic diameter (Dh), PDI, and ζ-potentials of NSs, the NS–antimicrobial complexes, AgNPs, and the ternary systems were provided by a DLS Zetasizer NanoS series, Malvern. Measurements were performed at 298 K after dilluting the samples with Milli–Q water. The results were reported as average after acquiring 12 measurements for each sample. The conditions of said measurements were chosen according to the systems; a refraction index of 1.49 and a k of 0 were set for β–CD-based samples, whereas a refraction index of 1.33 and a k of 3.99 were set for colloidal AgNPs samples.

### 2.7. Complexation Efficiencies of the NS–QRC Formulation

The encapsulation efficiency (EE) of NS–QRC complex was calculated from the absorbances of QRC and converted to concentration using UV–Visible spectrophotometry and the Lambert–Beer law. Further, Equation (1) was used to calculate the EE% of the system, as follows:

(1) $$EE\left(\%\right)=\frac{\left[Guest\right]\;in\;NSs}{\left[Guest\right]\;initially}\times 100\%$$

On the other hand, the loading capacities (LC) of NS–QRC were determined from the weights of NSs and the encapsulated antimicrobial using Equation (2):

(2) $$LC\left(\%\right)=\frac{QRC\;mass\;in\;NSs}{Mass\;of\;NSs}\times 100\%$$

### 2.8. Antibacterial Activity

The antibacterial effect of the NS–QRC and AgNP–NS–QRC complexes against Escherichia coli ATCC25922 was evaluated. First, bacteria were cultivated in tryptic soy broth for 24 h at 37 °C.

The culture’s optical density (600 nm) was adjusted to 1, at which the inoculum was estimated to have 5 × 10^8^ CFU/mL. Concentrations from 0.4 to 3.6 mg/mL of each sample were added to 96 well plates with tryptic soy broth as culture media and inoculated with 20 μL of the bacteria inoculum.

The plates were incubated at 37 °C for 24 h, and optical density at 600 nm was measured in a spectrophotometer (Tecan Infinite pro 200, Männedorf, Switzerland). Wells charged with bacterial inoculum were used as viability control, and assays with only AgNPs and QRC were also performed for comparison. Representative images of the antimicrobial activity assays are presented in Appendix E.

### 2.9. MTS Cell Metabolic Activity Assay

GES–1 cells were cultured in Dulbecco’s modified Eagle’s médium (DMEM), with phenol red, containing 1% penicillin/streptomycin and 10% fetal bovine serum. The culture was maintained at 37 °C and 5% CO_2_.

To determine the effect of the NS–QRC and AgNP–NS–QRC complexes on cell metabolic activity, an MTS assay was performed, which established of a linear relationship between the viable cell number and absorbance. The treatments were prepared from a standard 40 mg/mL solution in water containing DMSO (1% *v*/*v*). A vehicle control tretatmente (water + DMSO 1% *v*/*v*) was performed.

GES–1 cells (1 × 10^4^ cells/well) were seeded in pretreated 96 wells plates and allowed to attach at 37 °C, 5% CO_2_. The medium was removed, and a fresh medium containing the treatments was added and allowed to incubate for 24 h. After this time, cell metabolic activity was measured (in quadruplicate) in four independent experiments using the MTS assay according to manufacturer’s protocol. Subsequently, the absorbance of the culture medium at 490 nm was recorded and data were expressed as percentages of surviving cells (mean ± SEM). Representative images of MTS cell viability assays are presented in Appendix F.

### 2.10. Statistical Analysis

All reported results are the average ± SD, where all measurements were performed three times (*n* = 3). The statistical significance for the results (*** *p* < 0.001) was validated using GraphPad Prism 9 Software, Inc. (San Diego, CA, USA). The statistical tests and significant differences were determined utilizing a Dunnett test as a part of a one-way ANOVA.

## 3. Results and Discussion

### 3.1. Characterization of the ICs

#### 3.1.1. FE–SEM of the NS–QRC Complex

**Figure 3:**
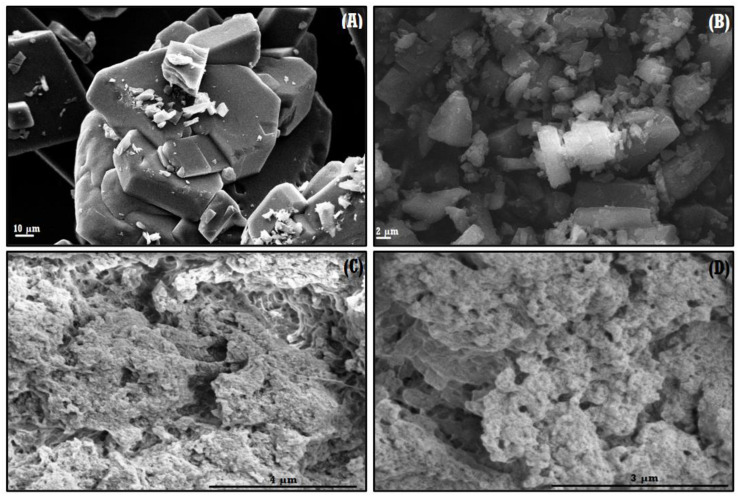
FE–SEM micrographs of QRC (**A**,**B**), NS–QRC (**C**,**D**) at different magnifications: 10,000× (**A**), 5000× (**B**), 60,000× (**C**,**D**).

The formation of the NS–QRC formulation can be confirmed by FE–SEM micrographs. The morphological features of the systems can be evaluated before and upon complexation and loading of the guests. Figure 3 shows SEM micrographs of QRC and the NS–QRC complex (for FE–SEM images of free NSs, see Appendix A).

As reported previously, FE–SEM images of NS–drug complexes depict a highly rough surface, suitable for the entrapment of guest molecules [36]. The rough morphology of the ICs seems to be uniform in the whole sample. On the other hand, QRC shows crystalline morphology. This suggests that the NS–QRC complex was formed, excluding the possibility of a physical mixture between the species. Complexing the NSs with QRC is an effective way to stabilize and preserve the guests’ stability, as the crystalline structure of free QRC was not observed in the formulation [18,37,38].

#### 3.1.2. TGA of the NS–QRC Complexes

**Figure 4:**
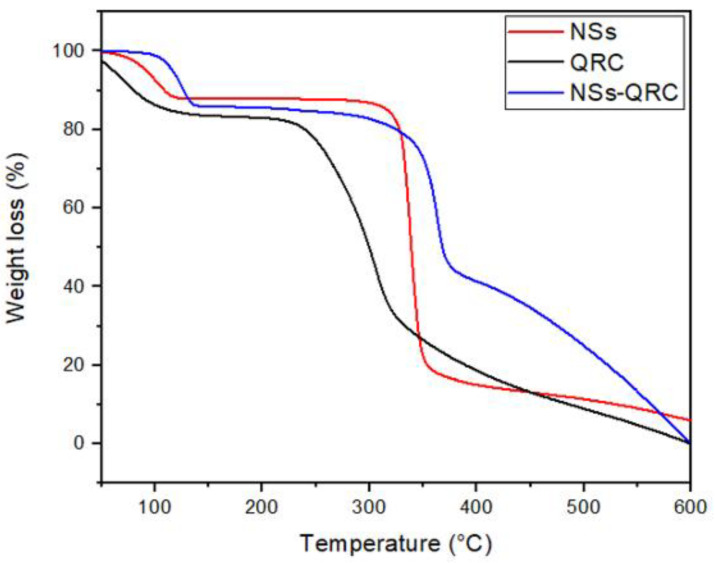
TGA of NSs, QRC, and the NS–QRC complex.

TGA curves of the free drug, free NSs, and ICs were performed and analyzed to differentiate the degradation steps of the species after the formation of the complexes. Figure 4 shows the TGA profiles of QRC, NSs, and the NS–QRC complex.

Free QRC showed a mass loss at 157 °C, which is consistent with the thermal profiles of hygroscopic samples, whereas the same weight loss can be observed in the TGA profile of free NSs (adapted from [28]). Maximum weight loss of QRC and NSs were observed in the second step of decomposition at about 313 °C and 350 °C, respectively. Notably, the main degradation step of the NS–QRC complex occurred at 370 °C, suggesting that the encapsulation of the guests provided thermal stability and prevented their degradation [11,39,40]. Table 1 summarizes the decomposition temperatures of all samples. Furthermore, the decomposition ranges discussed above were used to estimate the amount of QRC present in the NS–QRC (see Appendix B).

#### 3.1.3. XRPD of the NS–QRC Complexes

**Figure 5:**
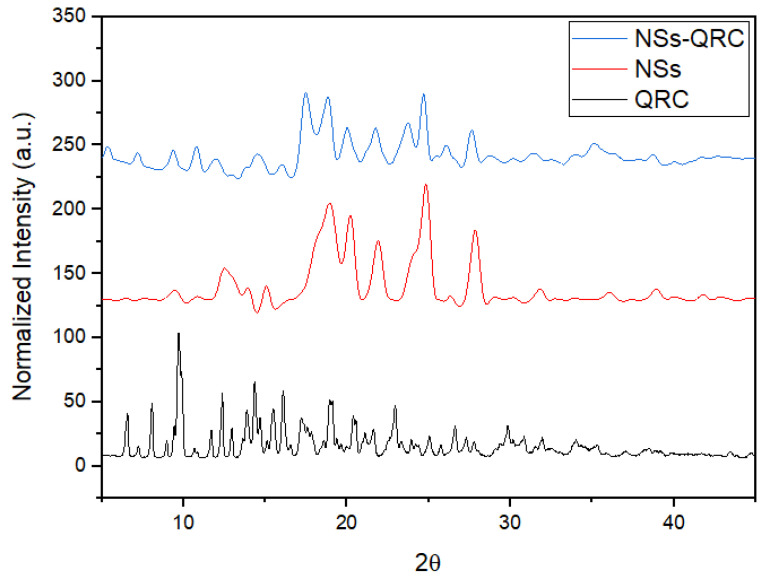
XRPD of NSs, QRC, and NS–QRC.

XRPD of NSs, QRC, and the NS–QRC complex were analyzed to ascertain the crystallinity of the free species and the developed formulation, as shown in Figure 5.

The diffractogram of free QRC shows its characteristic sharp diffraction peaks, thus evidencing its high crystalline nature. On the other hand, NSs instead displayed an amorphous form, which is consistent with that previously reported [35,36,37,41]. Further, the NS–QRC complex revealed that the sharp and characteristic peaks of QRC were drastically reduced upon inclusion in the NSs interstices, indicating the amorphization of the species. Notably, the free Gibbs energy of amorphous systems is higher than its crystalline state, thus enhancing its solubility and bioavailability [2,18,42]. Furthermore, the enhancement of the anti-microbial compounds’ solubility markedly increases their inhibition properties, in accordance with previous studies [16,17,18,35].

The relative degree of crystallinity (RDC%) of all systems was obtained from their diffraction patterns by comparing the area of the total crystalline phase with the total area of the crystalline and amorphous state, as follows (Equation (3)):

(3) $$RDC\left(\%\right)=\frac{IC}{IC+IA}\times 100\%$$

The RDC (%) of QRC, NSs, and NS–QRC is displayed on Table 2.

The RDC values suggest the host–guest interaction between the NSs and QRC, which promotes a decrease in the molecular order and an amorphous state of the formulation [18,36,37,42].

#### 3.1.4. Loading Capacity (LC%) and Encapsulation Efficiencies (EE%) of the NS–QRC Complex

By using Equations (1) and (2), the encapsulation efficiency (EE%) and the loading capacity (LC%) of the developed NS–QRC formulation can be estimated. As shown in Table 3, the amount of entrapped drug within the cavities of the NSs displayed increased values in compared with the native β–CD, suggesting that the structure of the guest might influence crucial factors such as complexation efficiency and host–guest interactions. The multiple hydrophobic inclusion and non-inclusion sites of NSs could be more aptly sized than β–CD to include QRC. The calculated EE% and LC% values for NS–QRC and β–CD–QRC are summarized in Table 3.

#### 3.1.5. ^1^H–NMR of the NS–QRC Complexes

**Figure 6:**
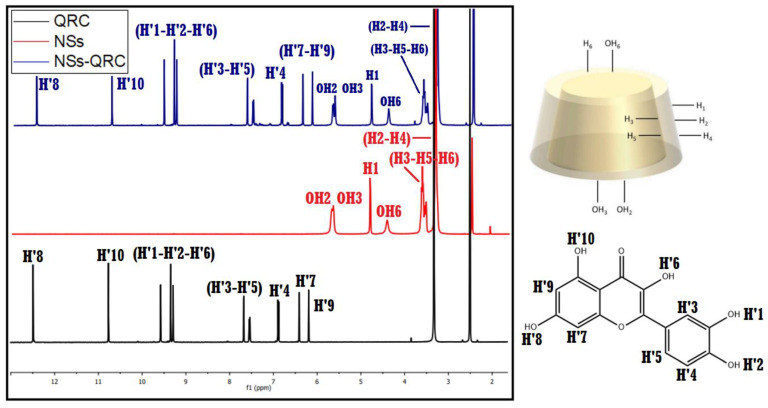
^1^H–NMR of NSs, QRC, and NS–QRC.

The inclusion and non-inclusion phenomena in the NS–QRC complex were confirmed using ^1^H–NMR spectroscopy. The chemical shifts in the protons of the species provide information for the interaction between the interstitial sites of NSs and the free guest. The recorded spectra of NSs, QRC and the NS–QRC formulation are shown in Figure 6.

High-field chemical shifts were observed in all the proton signals of QRC after the host–guest complex formation compared to free QRC, which can be ascribed to a change in its chemical environment and the spatial restriction of the antimicrobial. This strongly suggests the interaction of QRC with the NSs carrier [14,19,43].

Furthermore, NSs spectra upon inclusion demonstrated that the hydrophobic protons of NSs (H3, H5, and H6), as well as the protons in the outside cavities of the polymer (H2, H4, and H6) also displayed changes in their chemical shifts, evidencing the entrapment of QRC in both the β–CD monomers and the interstices within NSs produced in the polymerization [19,27,28].

The most pronounced chemical shifts were observed in the H3, H5, and H6 protons of the NSs, revealing the preferential inclusion of QRC in the lipophilic cavities present in the β–CD monomer. The hydroxyl groups of NSs exhibited downfield shifts, possibly due to a hydrogen-bonding phenomenon in the NS–QRC complex formation. These results are summarized in Table 4.

### 3.2. Characterization of NS–QRC Associated with AgNPs

#### 3.2.1. TEM, UV–Vis, XRPD, SAED, DLS, and ζ-Potential of AgNPs

**Figure 7:**
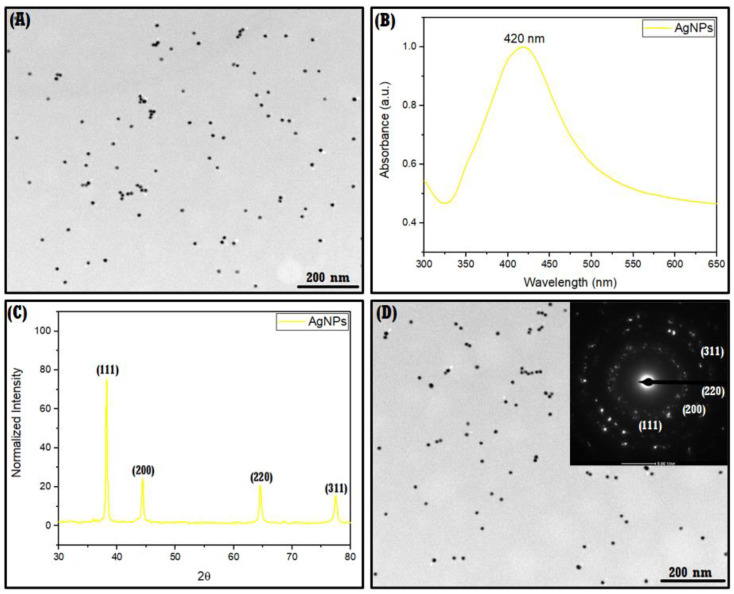
TEM (**A**), UV–Visible spectra (**B**), XRPD (**C**), and SAED (**D**) of AgNPs.

AgNPs before immobilization in the NS–QRC complex were characterized by TEM, XRPD, SAED, DLS, UV–Vis, and **ζ**-Potential, as shown in Figure 7. The average size distribution of AgNPs was estimated using TEM micrographs (Figure 7A), suggesting that the synthesized nanoparticles are monodispersed and exhibit an average size of 12 ± 2 nm. The UV–Vis spectrum (Figure 7B) of AgNPs confirms their plasmonic nature, as the characteristic plasmonic band of said nanoparticles shows a maximum absorbance peak at 420 nm [31,32]. The measurement of XRPD of AgNPs was analyzed in Figure 7C, where the obtained data showed diffraction peaks with the following indices: (111), (200), (220), and (311), which is consistent with the selected area electron diffraction (SAED) pattern shown in Figure 7D, where the lattice planes can be indexed to AgNPs crystalline structure [34,44].

The D_h_, the ***ζ***-Potential, and the polydispersity index of AgNPs are summarized in Table 5. DLS provided a hydrodynamic diameter of 31 ± 3 nm. The information obtained from the DLS corresponds to the size of the nanoparticles and the hydration sphere surrounding the metal, thus justifying the differences from the average size observed in TEM. A PDI of 0.21 indicates good monodispersity, which is consistent with the micrographs observed in Figure 7A. Furthermore, the measured ***ζ***-Potential of AgNPs was −33 ± 5 mV, indicating the colloidal stability of the AgNPs, which would prevent their agglomeration over time [44,45].

#### 3.2.2. TEM of the NS–QRC Complex Associated to the AgNPs

**Figure 8:**
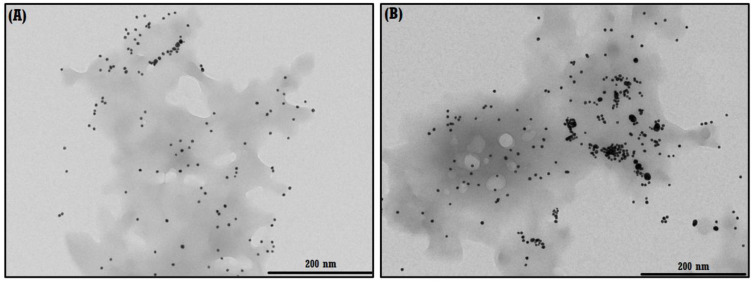
TEM micrographs of AgNP–NS–QRC. The AgNPs are both dispersed (**A**) and aggregated (**B**) in the NS–QRC formulations.

Due to the aqueous solubility of the ternary system, the analysis of the immobilization of AgNPs in the NS–QRC complex was conducted using TEM micrographs, which are shown in Figure 8A,B. The AgNPs maintain their integrity upon association with the NS–QRC formulation, confirming that the organic matrix is an appropriate substrate for nanoparticle stability.

#### 3.2.3. FE–SEM and EDS Analyses of NS–QRC Associated with AgNPs

**Figure 9:**
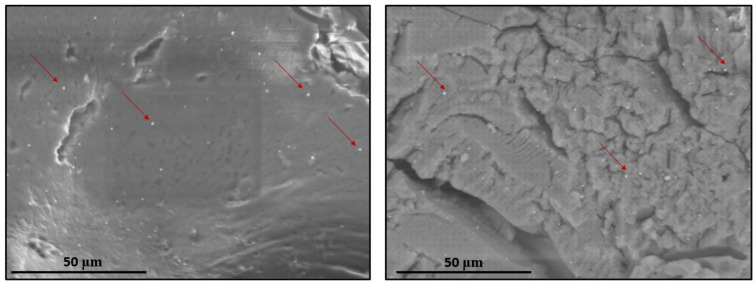
FE–SEM micrographs of AgNP–NS–QRC. Red arrows highlight the AgNPs deposited on both the smooth (**left** image) and rough (**right** image) surfaces of the NS–QRC complex.

FE–SEM characterization was employed to study the surface of the ternary complex. As indicated in Figure 9 the AgNPs are embedded all over the NS–QRC complex with no apparent changes in their spherical morphology, supporting the results described in Figure 8A,B. Moreover, no disruption of the surface of the NS–QRC complex was evidenced.

**Figure 10:**
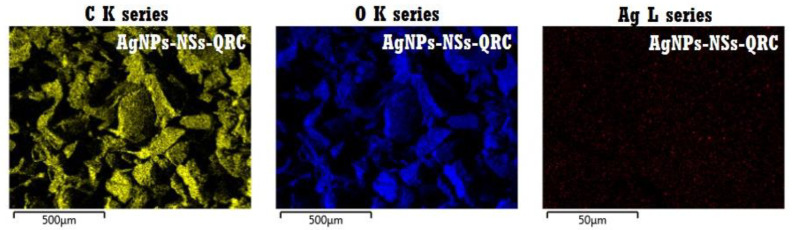
EDS mapping of AgNP–NS–QRC.

To prove the association of the NS–QRC formulation with AgNPs, an EDS elemental mapping analysis was also performed as shown in Figure 10. C and O were detected in the AgNP–NS–QRC complex, which can be ascribed to the hydroxyl and carbonyl functional groups present in the molecular structures of both NSs and QRC. The elemental mapping also evidenced the presence of Ag, suggesting the immobilization of AgNPs onto the NS–QRC formulation.

#### 3.2.4. UV–Visible Spectrum of NS–QRC Associated with AgNPs

**Figure 11:**
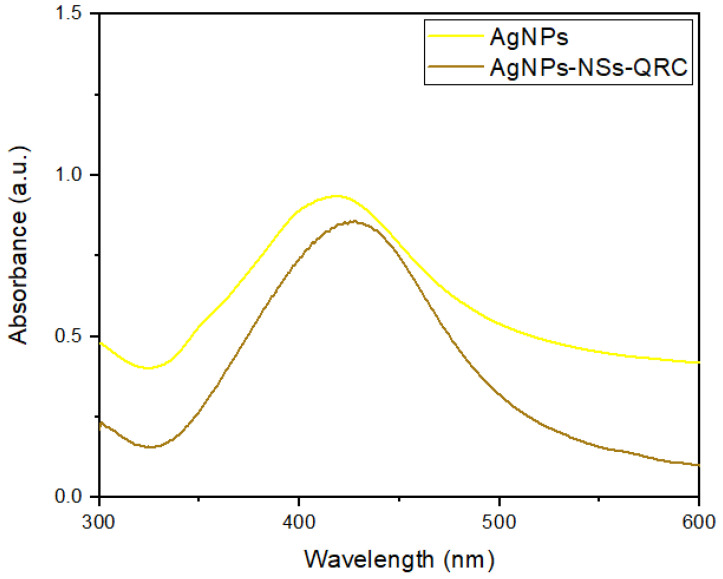
UV–Visible spectrum of free AgNPs and the AgNP–NS–QRC complex. A bathochromic shift from 420 to 430 nm was observed.

The immobilization of AgNPs onto the NS–QRC formulation can also be confirmed with UV–Visible spectroscopy. As described in Figure 11, the characteristic plasmonic band of AgNPs appeared at 430 nm. Both a bathochromic shift and a broadening of the plasmon band were observed, which could be attributed to the aggregation of some AgNPs upon association [28,29].

Nonetheless, the presence of the absorption band in the ternary systems confirms that the nanoparticles retain their integrity once deposited onto the organic matrix.

#### 3.2.5. DLS and ζ-Potential of NS–QRC Associated to the AgNPs

NSs, the NS–QRC complex, and the ternary system were also characterized using DLS, PDI, and ζ-potential as described in Table 6. DLS provided information regarding the D_h_ of all systems, which confirmed the nanometric sizes of the species, as NSs, NS–QRC, and AgNP–NS–QRC depicted values of 175, 233, and 261 nm, respectively.

The ζ-potential of free AgNPs decreased compared to the AgNPs deposited onto the NS–QRC complex, which is attributable to the stabilization of the nanoparticles by the inclusion compound. All nano-suspensions displayed ζ-potential values over ± 20 mV, which can be considered stable [46,47].

The obtained PDI values below 0.7 support the facts discussed above, confirming the stability and homogeneity of all the formulations (see Appendix C for further information).

### 3.3. Antibacterial Assays of NS–QRC Associated with the AgNPs

As reported in the literature, quercetin is known for its antibacterial properties that have great potential in both medical and pharmaceutical fields [16,17,18]. Therefore, the inclusion complexes’ antibacterial effects were analyzed to compare the activity of both NS–QRC, NS–AgNPs, NS–QRC associated with AgNPs, free QRC, and bare AgNPs. The different systems were tested by adding different volumes of a concentrated stock of each sample in 96 well plates containing bacterial broth and inoculating with *Escherichia Coli*. The study was completed under the same conditions using AgNPs, QRC, and NS–AgNPs as controls to ascribe the observed effects with the developed formulations, their compounds, and their stability. The percentages of inhibition of bacterial growth are shown in Table 7.

As previously mentioned, this substance inhibits bacterial growth, but the antibacterial activity observed in the assay is superior for NS–QRC than for free QRC. Regarding the control samples, the antibacterial capacity was lower for AgNPs and NS–AgNPs than for free QRC and the NS–QRC and AgNP–NS–QRC complexes. It can also be observed that the percentage of inhibition is higher for the NS–QRC formulation at the concentrations of 2.0, 2.8, and 3.6 mg/mL than for any other sample, including free QRC.

As the concentration of QRC is the same in all the assayed samples, the higher antibacterial capacity observed for NS–QRC over free QRC could be first attributed to the increased solubility of the guest in the complex, which might promote the diffusion of the active towards the bacteria in culture media [18]. It may also be a consequence of the gradual release of QRC from the NS matrix, allowing the conservation of QRC integrity and the proper exertion of its antibacterial activity. These could also be applied to the results obtained for the NS–QRC complex with AgNPs, in which the antibacterial effect is less than the observed for NS–QRC as the release of the guest from the complex is slower because of the high stability of the ternary complex [31,33], thus increasing the permanence of the antimicrobial in the site of action (the facts explained above are supported by the results shown in Appendix D).

On the other hand, the reduced antibacterial behaviour of AgNPs compared with NS–QRC and the ternary systems can be attributed to the agglomeration of bare AgNPs outside the microbial cell walls, as reported previously in similar studies [48].

### 3.4. Cell Metabolic Activity Assays

**Figure 12:**
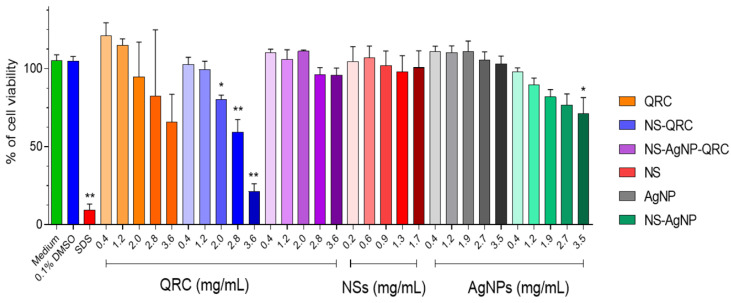
Cell metabolic activity assays in GES–1 cell line treated with NS–QRC, AgNP–NS–QRC complexes, as well as with the controls (QRC, NSs, AgNPs, and NS–AgNPs) at different concentrations. Data are represented as mean ± SEM of four independent experiments in quadruplicate. Statistical significance is indicated with * *p* < 0.01 or ** *p* < 0.0001.

To assess the impact of the inclusion complex on eukaryotic cell metabolic activity, an MTS assay with human gastric epithelial cell line (GES–1) was performed. GES–1 cells were chosen for their suitability as a human epithelial cell line. The effects of NS–QRC, NS–QRC with AgNPs, and free QRC depending on the concentration of QRC (tested at 0.4 to 3.6 mg/mL) were compared. In addition, the study also tested the cellular metabolic activity of GES–1 after treatment with AgNPs, NS–AgNPs, and NSs at equivalent concentrations to AgNPs (0.4 to 3.6 mg/mL) or NSs (0.2 to 1.7 mg/mL) as controls, as illustrated in Figure 12.

The results obtained showed that only the NS–QRC system depicts a significant reduction in cell metabolic activity at concentrations above 2.0 mg/mL. Figure 12 shows the metabolic activity was reduced to 20% at 2.0 mg/mL and a 40% at 2.8 mg/mL (Figure 12, blue bar), which can be considered as a slight and moderate cytotoxicity respectively [49]. However, the NS–QRC complex with AgNPs does not have adverse effects on cellular metabolism at the evaluated concentrations (Figure 12, purple bar), which are also observed for the AgNPs, NSs, and QRC separately (Figure 12, bars red, grey, green). This finding is highly relevant because it suggests that this ternary system does not affect the cellular metabolism but enhances the antibacterial activity of QRC (Table 7).

## 4. Conclusions

QRC was successfully included inside the supramolecular sites of NSs, proven by FE–SEM, TGA, XRPD, and ^1^H–NMR characterizations. This confirmed that NSs efficiently encapsulate QRC through host–guest interactions. The AgNPs were incorporated into the NS–QRC binary system. TEM, DLS, ζ-potential, UV–Vis, SAED, FE–SEM, and EDS provided evidence that the NS–QRC complex is an appropriate substrate to stabilize the AgNPs, retaining their characteristic absorption band and morphology. Results support the use of the system proposed and manufactured here to promote the guest compound’s stability and its antibacterial properties. Gradually releasing QRC from the NS matrix and conserving QRC integrity improves its antibacterial activity without compromising the cell metabolic activity of GES–1 epithelial cells. Therefore, the developed formulations present many opportunities for biological and antimicrobial applications. To our knowledge, this is the first time drug delivery systems consisting of AgNPs and NSs are being reported, where AgNPs could also promote the controlled release of the drugs due to local photothermia and laser irradiation.

## Figures and Tables

**Table 1:** Decomposition ranges of QRC, NSs, and the ICs.

Sample	Decomposition Temperature (°C)
QRC	157.9–177.3
QRC	305.3–313.8
NSs	352.7–353.1
NSs-QRC	355.9–369.6

**Table 2:** Relative degree of crystallinity of QRC, NSs, and the NS–QRC complex.

Sample	RDC (%)
QRC	83.3 ± 4.7
NSs	30.1 ± 2.3
NS–QRC	27.7 ± 5.9

**Table 3:** EE% and LC% of the β–CD–QRC and NSs–QRC systems.

Sample	Encapsulation Efficiency (%)	Loading Capacity (%)
β–CD–QRC	60.5 ± 2.2	5.77 ± 1.8
NS–QRC	88.1 ± 3.9	17.7 ± 3.5

**Table 4:** Chemical shifts for NSs, QRC, and the NS–QRC complex before and upon inclusion.

Sample	H1	H2	H3	H4	H5	H6	OH2	OH3	OH6	
NSs	4.823	3.298	3.627	3.360	3.577	3.653	5.703	5.677	4.447	
NSs–QRC	4.820	3.295	3.609	3.355	3.560	3.640	5.710	5.690	4.440	
Δδ	0.003	0.003	0.018	0.005	0.017	0.013	−0.010	−0.013	−0.007	
**Sample**	**H′1**	**H′2**	**H′3**	**H′4**	**H′5**	**H′6**	**H’7**	**H’8**	**H’9**	**H’10**
QRC	9.637	9.323	7.455	6.837	7.635	9.111	6.355	12.57	6.055	10.88
NSs–QRC	9.622	9.310	7.445	6.822	7.622	9.101	6.340	12.44	6.044	10.75
Δδ	0.015	0.013	0.010	0.015	0.013	0.010	0.015	0.013	0.011	0.013

**Table 5:** Hydrodynamic diameter, ζ-Potential, and PDI of AgNPs.

System	D_h_ (nm)	ζ-Potential (mV)	PDI
AgNPs	31 ± 3	−33 ± 5	0.21

**Table 6:** DLS, PDI, and ζ-potentials of the NSs, NSs-QRC and AgNPs-NSs-QRC systems.

Sample	D_h_ (nm)	ζ-Potentials (mV)	PDI
NSs	175 ± 18	−35 ± 2	0.22
NS–QRC	233 ± 5	−22 ± 3	0.33
AgNP–NS–QRC	261 ± 11	−28 ± 5	0.48

**Table 7:** Inhibition percentages of bacterial growth at different sample concentrations.

Concentration (mg/mL)	% Inhibition ± Standard Deviation				
	Sample				
	QRC	AgNPs	NS–AgNPs	NS–QRC	AgNP–NS–QRC
0.4	9.5 ± 2.7	10.5 ± 3.2	4.7 ± 3.1	3.9 ± 0.6	9.7 ± 1.7
1.2	16.2 ± 1.3	14.8 ± 1.8	8.5 ± 0.9	6.7 ± 2.9	15.7 ± 0.9
2.0	20.3 ± 1.2	17.4 ± 1.5	9.8 ± 0.6	39.1 ± 2.2	19.0 ± 2.7
2.8	27.5 ± 0.7	21.7 ± 3.2	15.2 ± 0.5	74.2 ± 0.8	26.0 ± 1.8
3.6	30.8 ± 0.7	26.3 ± 1.6	19.1 ± 0.8	85.0 ± 0.4	36.3 ± 1.9

**Figure A1:**
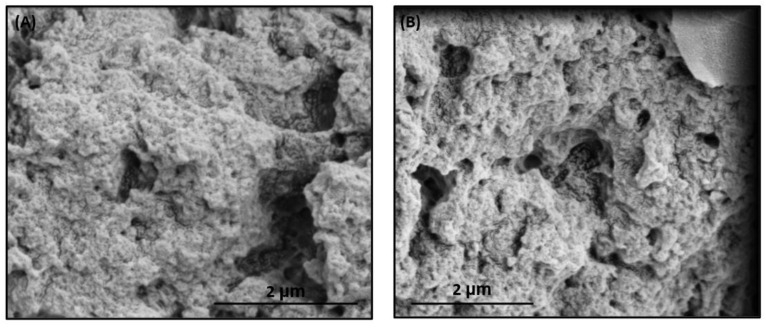
FE–SEM micrographs of the synthesized NSs at different magnifications: 60,000× (**A**), 50,000× (**B**).

**Figure A2:**
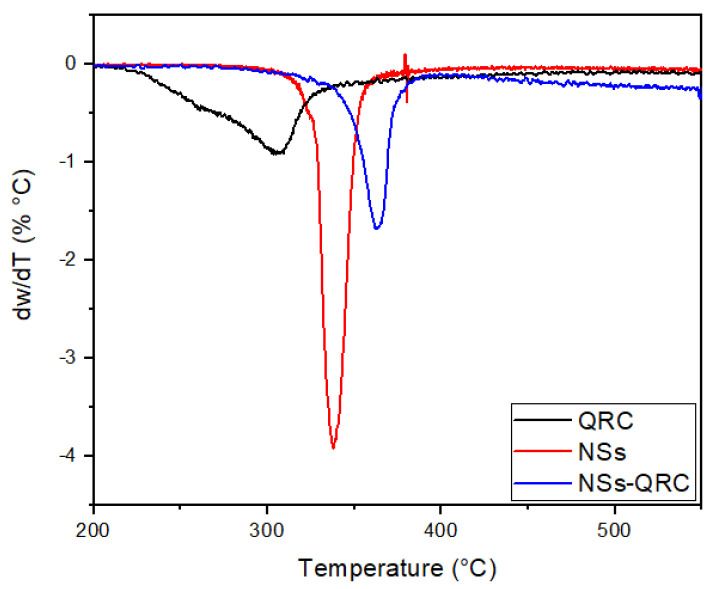
DTG curves of QRC, NSs, and NS–QRC.

**Figure A3:**
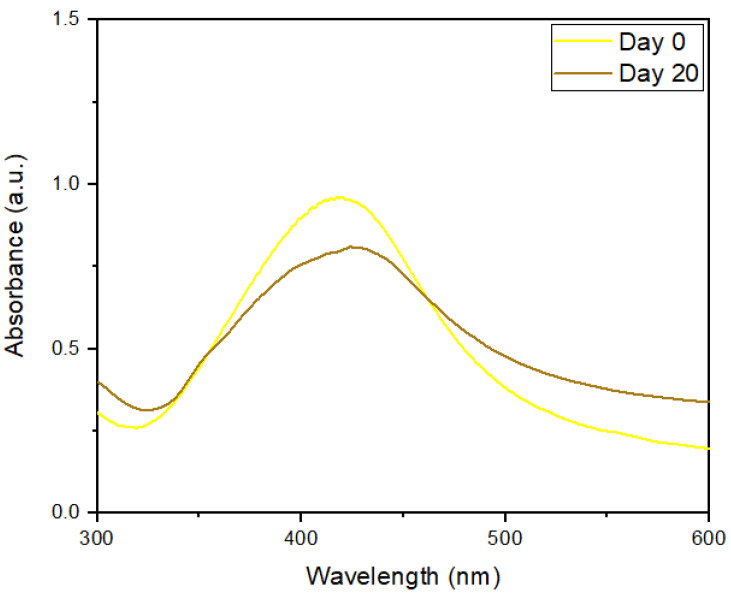
UV–Visible spectra of AgNP–NS–QRC following storage at room temperature (25 °C) for 20 days.

**Figure A4:**
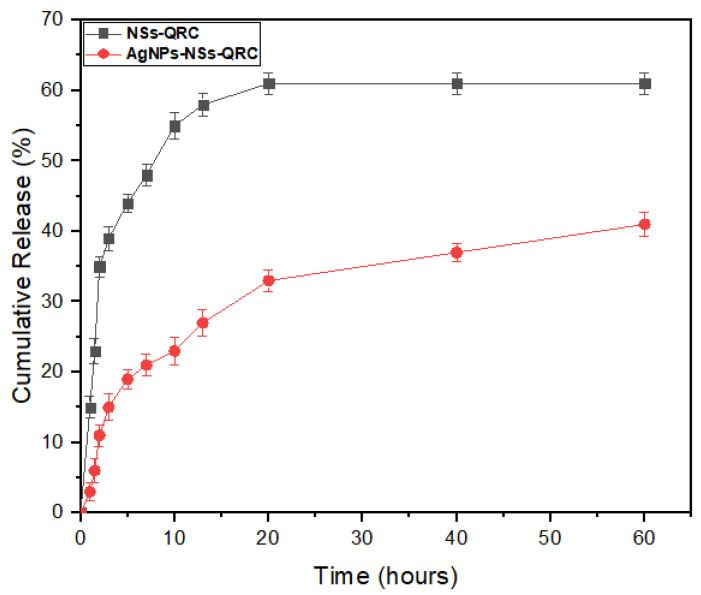
Drug release profiles of QRC from the supramolecular sites of NS–QRC and AgNP–NS–QRC.

**Figure A5:**
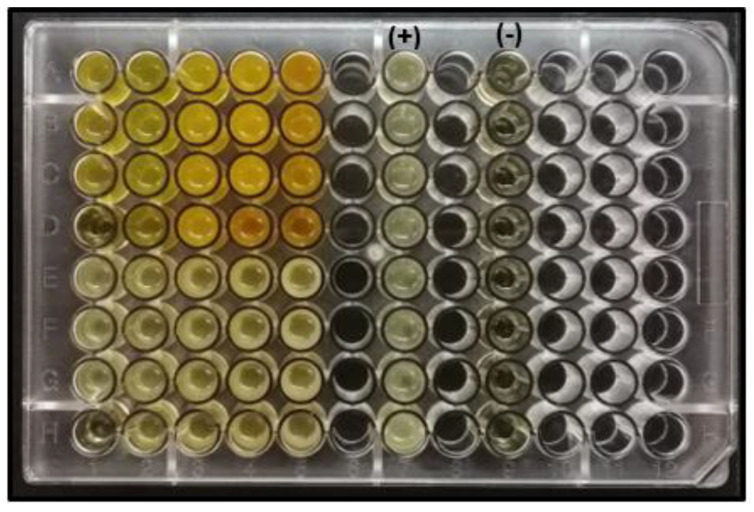
Antimicrobial activity assays (96 well plates) for AgNP–NS–QRC (A–D) and NS–QRC (E–H). The columns represent the increasing concentrations of the samples, where strong antimicrobial activity is evidenced by low turbidity. The (+) and (−) columns represent the positive control (culture media + bacteria inoculum) and negative control (culture media), respectively.

**Figure A6:**
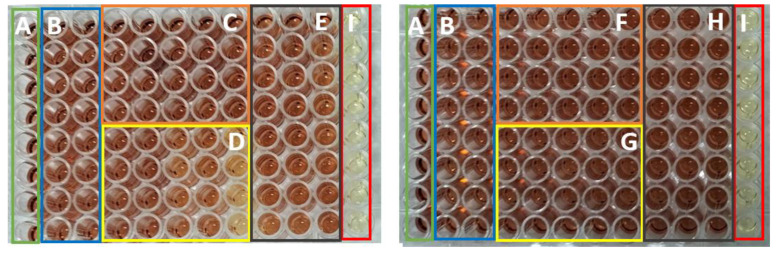
MTS representative assay (96 well plates) of GES–1 after 24-h treatment with QRC, NS–QRC, NSs (C–E) and AgNPs, NS–AgNPs, AgNP–NS–QRC (F–H). The columns represent the increasing concentrations of the samples, whereas the (A), (B), and (I) columns represent the medium (DMEM complete), vehicle (DMSO 1%), and death control (SDS), respectively.

## Data Availability

Not applicable.

## Appendix A

### FE–SEM Images of Free NSs

FE–SEM characterization evidenced the rough surface of the synthesized NSs, as observed in Figure A1. The morphology of free NSs does not significantly change upon the formation of the NS–QRC complex, thus preserving its sponge-like structure (see Section 3.1.2).

## Appendix B

### Determination of QRC Content Using Derivative Thermogravimetry (DTG)

The content of QRC present in the NS–QRC complex can be estimated using DTG, obtained by plotting the first derivative of the TGA measurements [50] shown in Section 3.1.2. Figure A2 shows free QRC showed a weight loss at 305 °C, whereas NSs and NS–QRC main decomposition steps were observed at 348 and 367 °C, respectively. From an additional weight loss in the NS–QRC matrix (−0.58 mg, initial mass of 3.11 mg), which was not observed in the weight loss profile of NSs (−1.322 mg, initial mass of 3.21 mg), the percentage of loaded QRC in the NS–QRC was determined, obtaining a loading capacity (LC%) of 18.65 ± 4.7%, which is in good agreement with the LC% calculated using UV–Visible spectrophotometry (see Section 3.1.4).

## Appendix C

### UV–Visible Spectra, DLS, and ζ-Potentials of AgNP–NS–QRC after 20 Days of Storage

The AgNP–NS–QRC ternary system was characterized through UV–Visible spectrophotometry, DLS, and ζ-potential to ascertain its colloidal stability following 20 days of storage at room temperature (25 °C). The characteristic surface plasmon band of AgNPs was still observed (Figure A3), suggesting that the NS–QRC formulation provides stability to the nanoparticles, even if not preserved at 4 °C, which is the recommended temperature to store and prevent the agglomeration of AgNPs [31,51]. Characterization utilizing DLS, PDI, and ζ-potential displayed that the ternary system maintained its nanometric dimensions, negative surface charge, and monodispersity (PDI > 0.7), as in Table A1.

**Table A1:** Hydrodynamic diameter, PDI, and ζ- potential of AgNP–NS–QRC.

Sample	D_h_ (nm)	ζ-Potentials (mV)	PDI
AgNP–NS–QRC (Day 0)	261 ± 11	−28 ± 5	0.48
AgNP–NS–QRC (Day 20)	273 ± 15	−23 ± 8	0.56

## Appendix D

### Cumulative Release of QRC from the NS–QRC and AgNP–QRC–NS Complexes

Cumulative QRC release from the NS–QRC and the ternary system was analyzed at 37 °C in a physiological medium (pH 7.4 in phosphate buffer saline, PBS). Neither of the release profiles displayed burst effects or drug leakage, which is relevant to prevent toxic side effects. Both profiles depicted a slow and sustained release of QRC over time, achieving a cumulative release of 60.3% ± 3.8 for NS–QRC and 40.1% ± 7.7 for AgNP–NS–QRC within 60 h. The lower cumulative release of QRC from the interstices in the ternary systems could be attributed to a slower diffusion of the released QRC due to the stability of incorporating AgNPs and its interaction with the carboxylic and hydroxyl functional groups of QRC [33,52].
